# Biofilm formation in *Streptococcus suis*: in vitro impact of serovars and assessment of coinfections with other porcine respiratory disease complex bacterial pathogens

**DOI:** 10.1186/s13567-024-01412-9

**Published:** 2024-12-01

**Authors:** Rubén Miguélez-Pérez, Oscar Mencía-Ares, César B. Gutiérrez-Martín, Alba González-Fernández, Máximo Petrocchi-Rilo, Mario Delgado-García, Sonia Martínez-Martínez

**Affiliations:** Department of Animal Health, Faculty of Veterinary Medicine, Universidad de León, León, Spain

**Keywords:** *Actinobacillus pleuropneumoniae*, *Glaesserella parasuis*, *Pasteurella multocida*, pig, respiratory pathogens, serotype, *Staphylococcus hyicus*, swine, virulence factor

## Abstract

*Streptococcus suis* is a worldwide pathogen that impacts the swine industry, causing severe clinical signs, including meningitis and arthritis, in postweaning piglets. A key virulence mechanism of *S.* suis is biofilm formation, which improves its persistence and resistance to external factors. Here, we assessed the in vitro biofilm formation of 240 *S. suis* isolates from Spanish swine farms and evaluated the effects of serovars (SVs) and coinfections with other porcine respiratory disease complex (PRDC) pathogens. Our study revealed significant heterogeneity in biofilm formation among *S. suis* SVs. Notably, SV2 resulted in the lowest degree of biofilm formation, in contrast with the high biofilm-forming capacities of SV1, SV7, and SV9. Other PRDC pathogens, including *Actinobacillus pleuropneumoniae*, *Glaesserella parasuis*, and *Pasteurella multocida*, formed biofilms, although they were generally less robust than those of *S. suis* (except for SV2), which contrasts with the high biofilm formation of *Staphylococcus hyicus*. Coinfections enhanced biofilm formation in mixed cultures of *S. suis*, particularly with *P. multocida*. Other coinfections revealed variable results in pathogen interactions, suggesting the potential of biofilms for increased persistence and pathogenicity in coinfections. In conclusion, this study underscores the importance of serovar-specific differences in biofilm formation among *S. suis* isolates, with significant implications for pathogenicity and persistence. The heterogeneous biofilm formation observed in coinfections with other PRDC pathogens reveals a complex interplay that could exacerbate disease severity. These findings provide a foundation for further research on biofilm mechanisms to mitigate the impact of PRDC in the swine industry.

**Supplementary Information:**

The online version contains supplementary material available at 10.1186/s13567-024-01412-9.

## Introduction

*Streptococcus suis* constitutes a worldwide hazard, not only because of its impact on the swine industry but also because it is a zoonotic pathogen [1]. During pig production, *S. suis* affects mostly postweaning piglets between four and ten weeks of age. Once the upper respiratory tract is colonized, the host usually becomes asymptomatic, eventually leading to the development of invasive disease, causing severe clinical signs, including arthritis, meningitis, endocarditis, septicaemia, and ultimately sudden death [2].

*S. suis* exhibits significant genetic and phenotypic heterogeneity, even among strains of the same serovar (SV). Currently, there are 29 well-defined SVs (119, 21, 2325, 2731, and 1/2) based on the antigenicity of the capsular polysaccharides [3]. Over one hundred virulence factors (VFs) have been described for *S. suis* in the literature [4]. However, despite the critical role of the capsular polysaccharide, no other specific VF has been definitively identified as essential for the disease [5]. Among them, we highlight the particular role of adhesins and cell surface factors, such as muramidase-released protein (MRP), extracellular factor (EF), and glyceraldehyde-3-phosphate dehydrogenase (GAPDH); toxins, such as suilysin; and S-ribosylhomocysteinase (LuxS), an interspecies quorum sensing-related enzyme [6].

Biofilm formation is a major pathogenic factor in *S. suis*, facilitating its establishment in pig tissues [7]. Biofilms are inter- or intraspecific communities of bacteria enclosed in a self-produced extracellular matrix that adheres to biotic or abiotic surfaces [8]. They have been linked to increased resistance to antimicrobial agents, environmental stress, and the host immune system, contributing to the chronicity of infections [9]. Although research on *S. suis* biofilms has been heavily promoted since its first description in 2007 [10], the understanding of their formation mechanisms remains superficial [7]. Moreover, little is known about the role of *S. suis* biofilm formation in coinfections with other bacterial pathogens directly or indirectly involved in the porcine respiratory disease complex (PRDC), a multifactorial syndrome affecting the respiratory system of postweaning piglets [11].

For these reasons, the aim of this study was to assess in vitro biofilm formation and characterize the VFs of 240 *S. suis* belonging to different SVs and recovered from Spanish pig farms, together with the evaluation of the in vitro biofilm formation capacity of a selection of bacterial pathogens frequently involved in the PRDC, alone and in coinfection with *S. suis.*

## Materials and methods

### Bacterial isolates and growth conditions

A wide range of different bacterial isolates were used in this study. First, 240 *S. suis* isolates belonging to 16 different SVs (i.e., 1, 2, 3, 4, 5, 7, 8, 9, 10, 12, 16, 17, 18, 19, 21 and 31) were isolated from three anatomic regions: the central nervous system (CNS), lungs and joints. For the other bacteria, a total of 35 virulent (*n*=21) and nonvirulent (*n*=14) *Glaesserella parasuis* belonging to eight different SVs (i.e., 1, 4, 5, 6, 7, 9, and 10), 31 *Staphylococcus hyicus*, 20 *Pasteurella multocida* belonging to five serogroups (i.e., A, B, D, E, and F), and 12 *Actinobacillus pleuropneumoniae* isolates belonging to 11 different SVs (i.e., 1, 2, 4, 5, 6, 7, 8, 9, 10, 11, and 12) were tested. A detailed summary of the isolates used in the study is available in Additional file 1. These isolates were recovered from clinical cases from Spanish swine farms collected between 2020 and 2024 and further included in the strain collection of the BACRESPI research group at the Animal Health Department of the University of León (Spain).

*S. suis* isolates were cultured on Todd-Hewitt broth (THB) agar (Condalab, Spain) supplemented with 5% (v/v) foetal bovine serum (FBS) (Gibco, USA) and grown at 37  for 24 h under aerophilic conditions. The remaining bacterial isolates were cultured on chocolate agar plates with Vitox (Oxoid, UK). *S. hyicus* and *P. multocida* were incubated at 37 °C for 24 h under aerophilic conditions, whereas *G. parasuis* and *A. pleuropneumoniae* were incubated at 37 °C for 48 h under microaerophilic conditions.

### Molecular characterization of bacterial isolates

Serotyping and virulence-related characterization of the different bacterial species were accomplished via polymerase chain reaction (PCR). *S. suis* characterization was based on the protocol described by Petrocchi-Rilo et al. [12], which included various sets of multiplex PCRs to identify all SVs and five monoplex PCRs for virulence-associated genes, including *epf*, *gapdh*, *luxS*, *mrp*, and *sly*.

For *S. hyicus*, multiplex PCR was used for the detection of genes encoding the exfoliative toxins ExhA, ExhB, ExhC and ExhD, as described by Andresen and Ahrens [13]. Six of the most relevant virulence-associated genes of *P. multocida*, namely, *hgbA*, *ompH*, *nanH*, *sodA*, *oma87*, and *pfhA*, were detected via PCR, as specified by Ewers et al. [14]. Another multiplex PCR method was used to differentiate the serogroups of *cap* genes A, B, D, E and F, as described by Townsend et al*.* [15]. For the *G. parasuis* isolates, PCR was utilized to classify the isolates into virulent or nonvirulent strains on the basis of *vtaA* genes, as described by Galofré-Milà et al. [16].

### Biofilm formation assay with single and mixed cultures

The biofilm formation of all the different isolates was quantified by crystal violet staining, following an archetypical biofilm formation protocol previously described [17] with slight modifications. Briefly, for *S. suis*, *S. hyicus* and *P. multocida*, a single colony was inoculated into 96-well polystyrene microfibre cell culture-treated plates (Corning Incorporated, USA) containing 200 µL of THB supplemented with 5% FBS. In the case of mixed cultures, a single colony of *S. suis* was inoculated, followed by inoculation of a single colony of either *S. hyicus* or *P. multocida*. For both single-colony and mixed cultures involving these pathogens, the plates were incubated for 24 h under aerophilic conditions. For plates containing either single *A. pleuropneumoniae* and *G. parasuis* isolates or mixed cultures with *S. suis*, THB was supplemented with 5% FBS, 0.5% glucose (v/v) and 20 mg/mL NAD, and the mixture was incubated for 48 h under microaerophilic conditions.

In either case, following incubation, the culture medium and unattached bacteria were aspirated to remove them. The formed biofilms were stained with 100 L of 2% crystal violet for 30 min, washed three times with distilled water, and dried at 37 °C for 15 min. To release the dye, 100 L of 95% ethanol was added, and the plates were briefly agitated. The absorbance of the biofilm biomass was quantified at 595 nm (A595). All the assays were conducted in triplicate to ensure the reliability of the results. The final optical density (OD) value of each isolate was expressed as the mean of the three measurements minus the average OD of the negative control (different from the control, DC) to reduce the possible unevenness in absorbance quantification.

### Scanning electron microscopy (SEM) of biofilms

The SEM assay was conducted on a selection of four *S. suis* isolates belonging to the four main SVs (SV1, SV2, SV7, and SV9) and a selection of two mixed infections of each bacterial pathogen evaluated with *S. suis*. It was performed following a previously published method [18] with minor adjustments. Briefly, Thermanox plastic coverslips (13 mm in diameter and 0.13 mm in nominal thickness; NUNC, USA), which were positioned at the bottom of the wells in 24-well polystyrene microfibre cell culture-treated plates (Corning Incorporated, USA), were used as adhesion carriers for the biofilms. Independently from the culture and after the appropriate incubation time (with all the media, supplement volumes and number of colonies proportionately extrapolated), the samples were fixed in 2.5% glutaraldehyde in phosphate buffer (0.1 M PBS, pH 7.4) at 4 °C for 12 h, rinsed three consecutive times with PBS, fixed with 1% osmium tetroxide in PBS for 45 min in the dark and rinsed again three times with PBS. The samples were dehydrated in a graded ethanol series (30%, 50%, 70%, 90%, 3×96%, and 3×100%, each for 10 min), dried by the critical point method (CPD300, Leica, Austria), mounted on aluminium stubs with a carbon ribbon and sputter-coated with goldpalladium (ACE200, Leica, Austria). The samples were observed under a Jeol JSM-6840LV scanning electron microscope (Jeol, Japan) at 5 kV. The samples were first approached via broad sweep visualization to further scrutinize representative areas to obtain images at either 2500X, 5000X or 10 000X magnification.

### Data analysis and results visualization

Databases were created in several Excel sheets (Microsoft Office). The first database, used for *S. suis* characterization and biofilm formation evaluation, included *S. suis* ID, anatomic location (lung, joint, CNS), SV, presence/absence of VF (*epf*, *mrp*, *sly*, *luxS*, and *gapdh*), and biofilm formation. Biofilm formation was expressed numerically as DC and categorized on the basis of DC value into low (DC2), medium (2>DC3), and high (DC>3) categories, as previously described [19].

The second database included the four most clinically relevant *S. suis* SVs (SV1, SV2, SV7, and SV9) and four additional bacterial pathogens also frequently associated with *S. suis* infections (*S. hyicus*, *P. multocida*. *G. parasuis* and *A. pleuropneumoniae*). In addition to the information from the first database, specific details were recorded for each microorganism: the VFs (*exhA*, *exhB*, *exhC*, and *exhD*) for *S. hyicus*; the VFs (*hgbA*, *ompH*, *sodA*, *pfhA*, and *oma87*) and capsular type (A, B, D, E, and F) for *P. multocida*; and the virulence (virulent/nonvirulent isolate) for *G. parasuis*. Coinfections were studied in a specific database for each pathogen pair, including all previous information along with biofilm formation in each coinfection.

Statistical analyses of DC values were conducted via nonparametric methods due to the nonnormal distribution of the data. Differences were assessed via the Wilcoxon rank-sum test, with *p* values adjusted according to the Benjamini and Hochberg method, and significance was established at *p*<0.05. Analyses for each bacterial pathogen were initially performed for all the isolates and further itemized by SV, anatomic location, or any other specific variables. *S. suis* isolates belonging to SV18 (*n*=1), SV19 (*n*=1), and SV31 (*n*=1), and non-typified isolates (*n*=7) were excluded from the statistical analysis because of their low frequency.

The *S. suis* isolates were estimated on the basis of their VF composition via the Jaccard distance matrix and analysed via principal component analysis (PCA). The two main dimensions of the principal components were characterized. The effect of biofilm formation capacity (low, medium, or high) was determined via permutational multivariate analysis of variance (PERMANOVA) using distance matrices with the *adonis2* function (pairwise adonis**)**.

For the evaluation of coinfections, four isolates belonging to the four most clinically relevant *S. suis* SVsSV1 (ID 990), SV2 (ID 1001), SV7 (ID 998), and SV9 (ID 969)were selected. These isolates were compared with a selection of ten random isolates from *S. hyicus*, *P. multocida*, *G. parasuis* and *A. pleuropneumoniae.* All analyses were performed within each *S. suis* SV and bacterial pathogen. To determine the effect of *S. suis* SV on the biofilm formation of each bacterial pathogen, the average DC of the bacterial pathogen was compared with the average DC of the coinfection with the specific *S. suis* SV. Similarly, to determine the effect of the bacterial pathogen on the biofilm production of *S. suis* SV, the average DC of the specific *S. suis* was compared with the DC of the coinfection with the pathogen.

All analyses were conducted using R version 4.3.2 (2023-10-31 ucrt) [20]. Plots were produced using the *ggplot2* package version 3.5.1 [21] and further modified using the software *Inkscape* version 1.3.2 [22].

## Results

### Characterization of *Streptococcus suis* clinical isolates on the basis of their virulence, serotyping, and anatomic location

Most isolates carried at least one VF, with frequencies of all VFs exceeding 60%. The most common VF was *luxS* (90%), followed by *gapdh* (80%), *epf* (64.6%), *mrp* (64.2%) and *sly* (62.5%). When the combinations of all VFs were evaluated, 27 distinct patterns were observed. The most frequent combination was the presence of all genes (33.8%), followed by the combination of *epf*, *sly*, *lux*S and *gapdh* and the combination of *luxS* and *gapdh*, each representing 8.3% of all *S. suis* isolates. A detailed description of VF patterns, both individually and by SV, is available in Additional files 2 and 3.

By analysing the isolates by SV, we found significant associations between certain SVs and VFs. Notably, there was a positive association of *epf*, *sly* and *mrp* with SVs 1, 2 and 9 (*p*<0.001) and a negative association of *epf* and *sly* with SV7 (*p*<0.05). Both *luxS* and *gapdh* were significantly more common in most isolates, regardless of SV, due to their high prevalence in the isolates of the study. A detailed description of these combinations is available in Additional file 4. Regarding *S. suis* anatomic location, we could determine that the only significant finding was a negative association between SV2 and the lungs (*p*<0.01), as most SV2 isolates were recovered from the CNS.

### Influence of serovars and virulence factors on the biofilm formation capacity of *Streptococcus suis*

**Figure 1:**
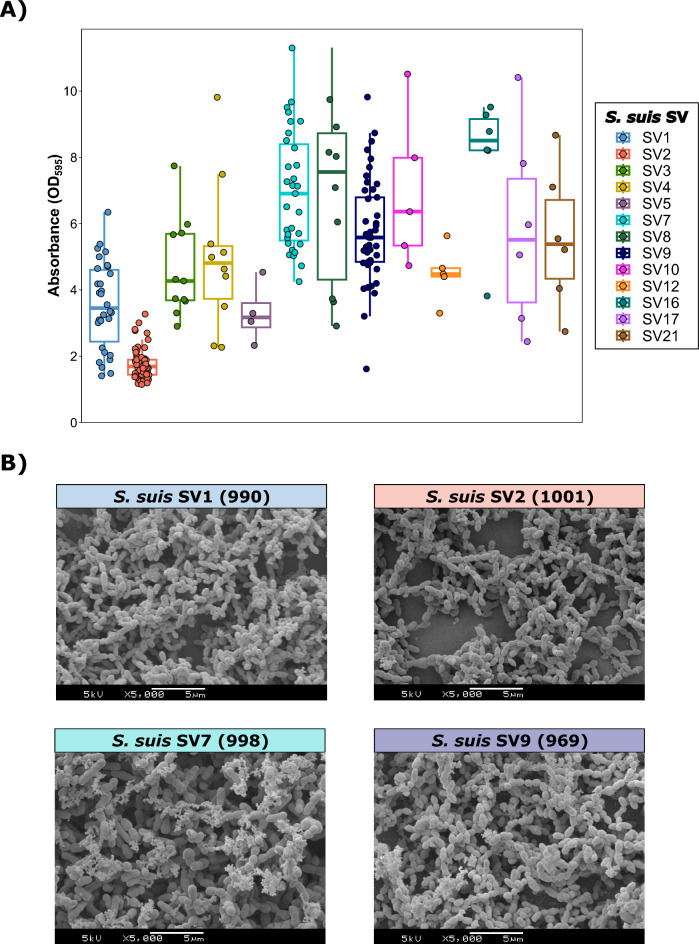
**Biofilm formation of *Streptococcus suis* isolates recovered from Spanish swine farms.** **A)** Boxplots illustrating biofilm formation categorized by *S. suis* serovar (SV). Biofilm formation for each isolate was quantified as the difference in absorbance (OD_595_) from that of the negative control (DC). Each *S. suis* isolate is represented by a dot with horizontal jitter for visibility. The horizontal box lines represent the first quartile, the median, and the third quartile. Whiskers extend to the 1.5 interquartile range. **B)** Scanning electron microscopy (SEM) images of biofilm formation in *S. suis* isolates belonging to the four main SVs (ID): *S. suis* SV1 (990), *S. suis* SV2 (1001), *S. suis* SV7 (998), and *S. suis* SV9 (969).

SV strongly influences the capacity of *S. suis* to produce biofilms (Figure 1A; Table 1). The most remarkable finding was the significantly lower biofilm formation of SV2 (DC=1.77±0.46) than that of most of the SVs commonly isolated from swine streptococcal infections (*p*<0.05), demonstrating its low biofilm formation capacity (DC2), as shown in the SEM images (Figure 1B). In contrast, other clinically relevant *S. suis* SVs, such as SV7 (DC=6.98±2.33) and SV9 (DC=5.77±1.56), presented strong biofilm formation capacity (Figure 1B). Overall, the mean biofilm formation for all the SVs, except SV2, was categorized as strong (DC>3). A detailed summary of the comparisons of biofilm formation capacity among SVs is available in Additional file 5. No significant association was detected between the anatomic location of *S. suis* and its biofilm formation capacity.

**Table 1:** Biofilm formation in ***Streptococcus suis*** isolates recovered from Spanish swine farms.

*S. suis* SV	Number of isolates (*n*)	Biofilm formation (mean±SD)	Biofilm formation (Degree)
SV1	30	3.54±1.32	High
SV2	61	1.77±0.46	Low
SV3	11	4.64±1.46	High
SV4	10	4.99±2.28	High
SV5	4	3.30±0.92	High
SV7	31	6.98±1.78	High
SV8	10	6.96±2.82	High
SV9	45	5.77±1.56	High
SV10	5	6.99±2.33	High
SV12	5	4.49±0.83	High
SV16	6	7.97±2.10	High
SV17	6	5.81±2.97	High
SV21	6	5.55±2.12	High

**Figure 2:**
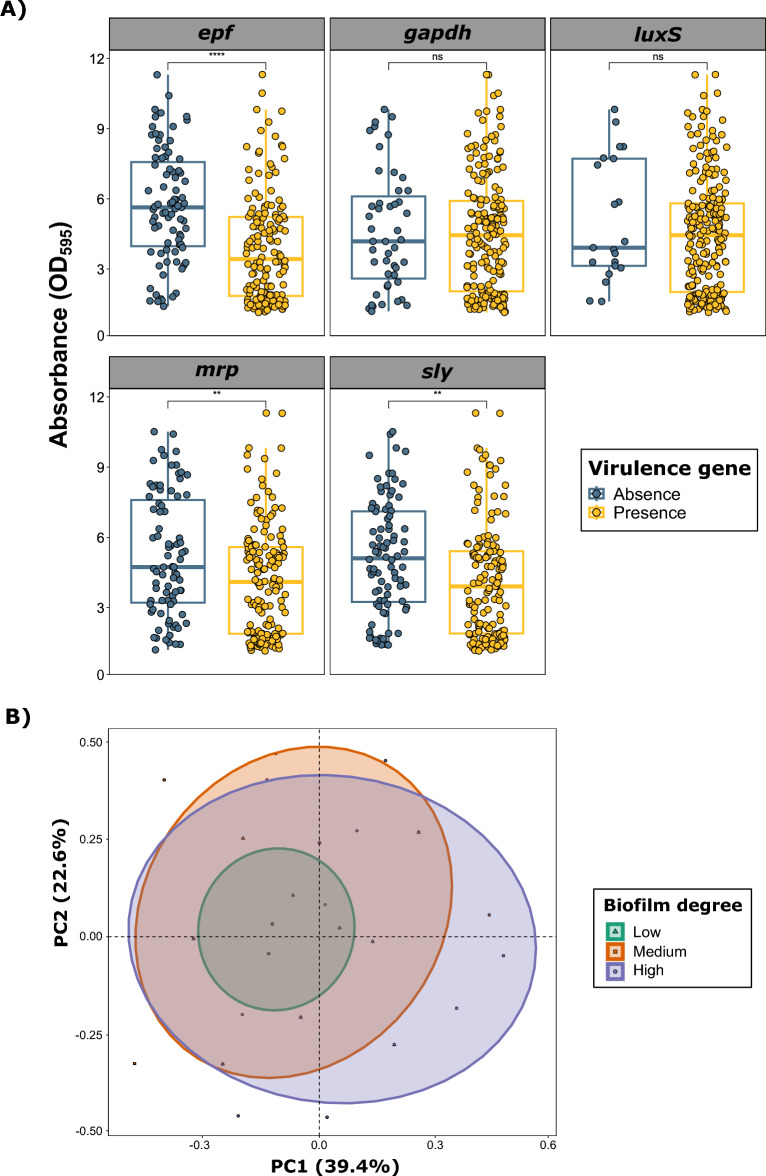
**Impact of virulence factor (VF) genes on biofilm formation in *Streptococcus suis* isolates recovered from Spanish swine farms.** **A)** Boxplots illustrating the quantitative biofilm formation of *S. suis*, comparing the presence or absence of VF genes. The difference in absorbance (OD_595_) from that of the negative control (DC) was quantified. Each *S. suis* isolate is represented by a dot with horizontal jitter for visibility. The horizontal box lines represent the first quartile, the median, and the third quartile. Whiskers extend to 1.5 times the interquartile range. Differences between groups were evaluated using the Wilcoxon rank-sum test. **B)** Principal component analysis (PCA) of the five evaluated VF genes, showing grouping on the basis of biofilm formation degree.

The analysis of the association between biofilm formation and VF composition (Figure 2A) revealed that isolates carrying *epf* (*p*<0.0001), *mrp* (*p*<0.01), or *sly* (*p*<0.01) genes had a lower biofilm formation capacity than those not carrying these genes. No significant differences were detected for *gapdh* and *luxS*. When evaluating the association between the degree of biofilm formation and VF combinations, we observed a slight (R^2^=0.03) but significant association (*p*<0.001) using PERMANOVA analysis (Figure 2B), with a slightly lower variation in VF composition among isolates with low biofilm formation capacity (DC2).

When the information is itemized by SV, the results vary. For example, SV1 isolates carrying the *epf* gene presented significantly higher biofilm formation (*p*<0.01), whereas SV2 isolates with the *epf* gene (*p*<0.05), SV9 isolates with the *sly* gene (*p*<0.01), and SV1 isolates with the *gapdh* gene (*p*<0.05) presented lower biofilm formation capacities.

### Biofilm formation in swine bacterial pathogens associated with *Streptococcus suis* infections

**Figure 3:**
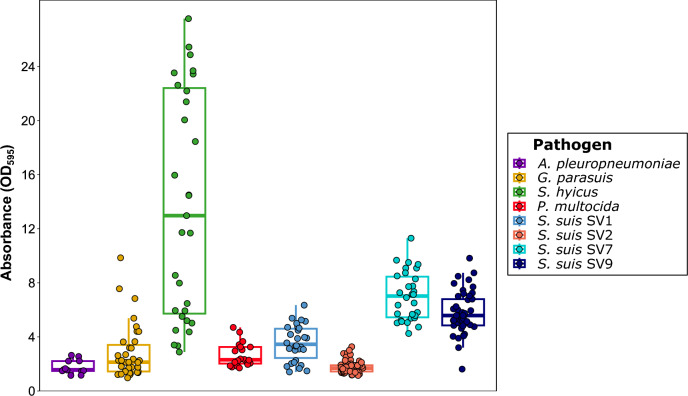
**Biofilm formation of bacterial pathogens involved in the porcine respiratory disease complex (PRDC) and *Streptococcus suis* isolates belonging to the four main serovars (SVs).** Boxplots illustrating biofilm formation for each isolate, quantified as the difference from the negative control (DC) in absorbance (OD_595_). Each isolate is represented by a dot with horizontal jitter for visibility. The horizontal box lines represent the first quartile, the median, and the third quartile. Whiskers extend to 1.5 times the interquartile range.

Substantial variability in biofilm production was observed among those pathogens frequently associated with *S. suis* infections (Figure 3). *S. hyicus* presented the highest biofilm formation capacity (DC=13.91±8.51) among all the tested species and *S. suis* SVs (*p*<0.001), despite notable variability among the isolates. It was followed by *S. suis* SV7 (DC=7.02±1.80), SV9 (DC=5.77±1.56), and SV1 (DC=3.54±1.32). *P. multocida* (DC=2.83±2.01) and *G. parasuis* (DC=2.66±0.88) were categorized as medium biofilm producers (DC>23). *A. pleuropneumoniae* (DC=1.76±0.51) and *S. suis* SV2 (DC=1.76±0.45) presented the lowest biofilm formation capacities. Interestingly, although *G. parasuis*, *P. multocida* and *A. pleuropneumoniae* produced significantly fewer biofilms than *S. suis* SV1, SV7 and SV9 did (*p*<0.05), the two former strains had significantly greater biofilm production than *S. suis* SV2 did (*p*<0.05). A detailed summary of biofilm formation capacity comparisons among bacterial species and *S. suis* SVs is available in Additional file 6.

We further evaluated additional information on these bacterial pathogens, revealing that no significant associations were observed between biofilm production and VFs in *S. hyicus*, VFs and the capsular type in *P. multocida*, or virulence in *G. parasuis*. For coinfection studies, we assessed the biofilm formation capacity of *S. suis* under microaerophilic conditions at 37 °C for 48 h, replicating *A. pleuropneumoniae* and *G. parasuis* growth conditions, and compared it with that under standard growth conditions (37 °C for 24 h under aerophilic conditions, optimal for *P. multocida* and *S. hyicus*). Given the clear differences between growth conditions, significant differences in biofilm formation capacity were observed, with greater production under microaerophilic conditions (*p*<0.05) also due to supplementation with glucose [23]. This finding does not interfere with further analyses, as coinfections were conducted individually for each pair of pathogens under the optimal growth conditions for the most fastidious microorganism.

### In vitro biofilm formation in coinfections between *Streptococcus suis* and clinically relevant swine bacterial pathogens

**Figure 4:**
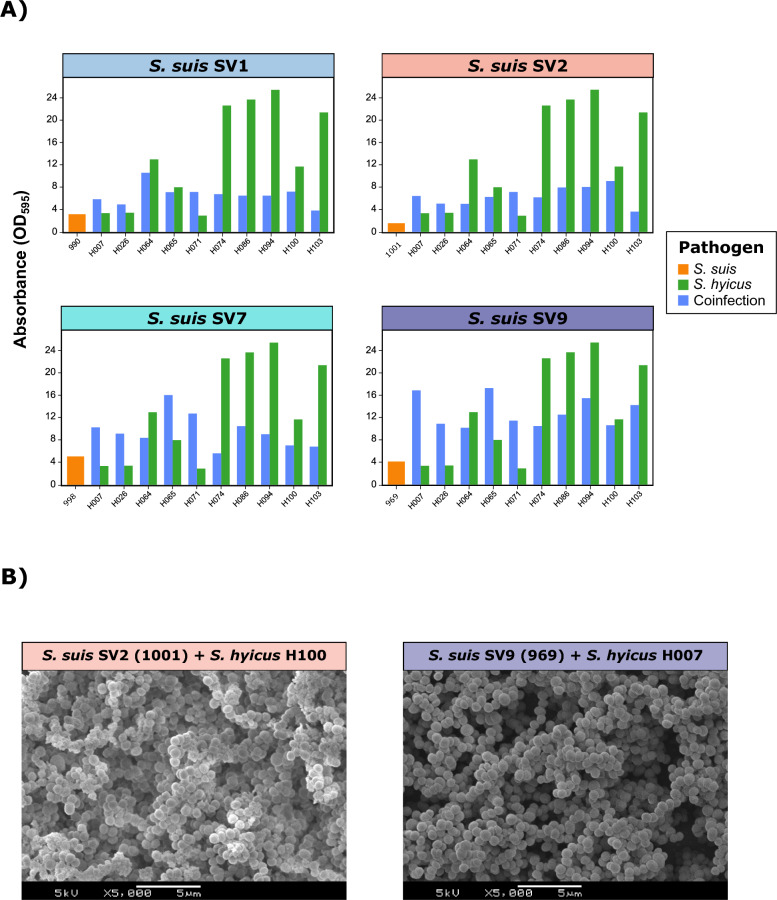
**Biofilm formation in coinfections of *Streptococcus suis* and *Staphylococcus hyicus*.** **A)** Bar plots showing the quantitative biofilm formation (expressed as the absorbance OD_595_) of one representative *S. suis* isolate from each of the four main serovars (SVs) (orange), ten random *S. hyicus* isolates (green), and their coinfections (blue). **B)** Scanning electron microscopy (SEM) images of biofilm formation in coinfections between *S. suis* SV2 (1001) and *S. hyicus* H100 (left) and between *S. suis* SV9 (969) and *S. hyicus* H007 (right).

Notable differences were observed among *S. hyicus* isolates and *S. suis* SVs during coinfection (Figure 4A). Interestingly, those *S. hyicus* strains with the highest biofilm production, specifically H074, H086, H094, and H103, exhibited lower biofilm formation when coinfected with *S. suis*, regardless of the SV. In contrast, isolates with lower biofilm formation, such as H007, H026, H065, and H071, demonstrated a synergistic effect with *S. suis* coinfection, which was particularly remarkable when coinfected with SV9 and SV7. SEM revealed that the increased biofilm formation observed for *S. suis* in these coinfections was caused mainly by *S. hyicus*, with a reduced presence of *S. suis*, regardless of the *S. suis* SV (Figure 4B). When *S. suis* SV was coinfected with *S. hyicus*, no significant differences were detected in the biofilm production of *S. hyicus*, but we observed a significant potentiation of biofilm formation in all *S. suis* SVs (*p*<0.0001), nearly doubling the DC value in coinfections with SV1 and SV7, 3.2 times for SV9, and 4.2 times for SV2.

**Figure 5:**
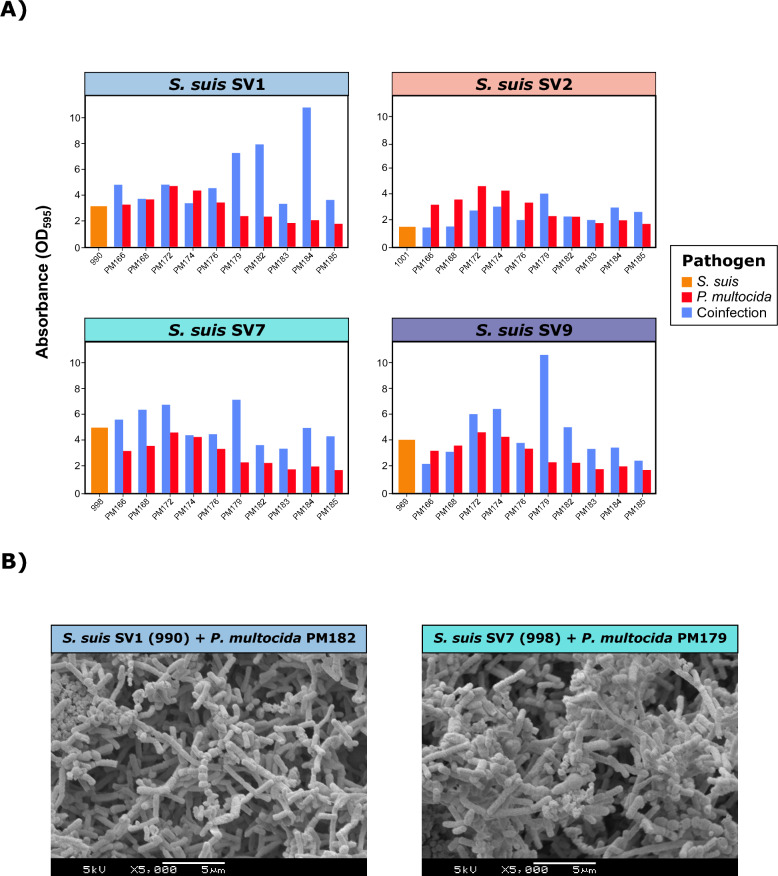
**Biofilm formation in coinfections of *Streptococcus suis* and *Pasteurella multocida*.** **A)** Bar plots showing the quantitative biofilm formation (expressed as the absorbance OD_595_) of one representative *S. suis* isolate from each of the four main serovars (SVs) (orange), ten random *P. multocida* isolates (green), and their coinfections (blue). **B)** Scanning electron microscopy (SEM) images of biofilm formation in coinfections between *S. suis* SV1 (990) and *P. multocida* PM182 (left) and between *S. suis* SV7 (998) and *P. multocida* PM179 (right).

Synergistic biofilm production was demonstrated in coinfections between *S. suis* and certain *P. multocida* isolates, especially for PM179 with all the SVs and, to a lesser extent, for PM182, except for SV2 (Figure 5A). With respect to the effect of *S. suis* SV, an increase in biofilm formation in *P. multocida* isolates was noted when coinfected with SV7 (*p*<0.01), nearly doubling the DC value, with a slightly greater contribution of *P. multocida* to biofilm formation (Figure 5B). A synergistic effect of *P. multocida* coinfection was demonstrated for SV2, increasing the number of *S. suis* SV2 DCs from 1.55 to 2.53 (*p*<0.01). Notably, a potentiation was observed for both *P. multocida* (*p*<0.01) and *S. suis* (*p*<0.0001) in SV1 coinfection, increasing to a DC of 5.42 from 2.97 and 3.13, respectively (Figure 5B).

**Figure 6:**
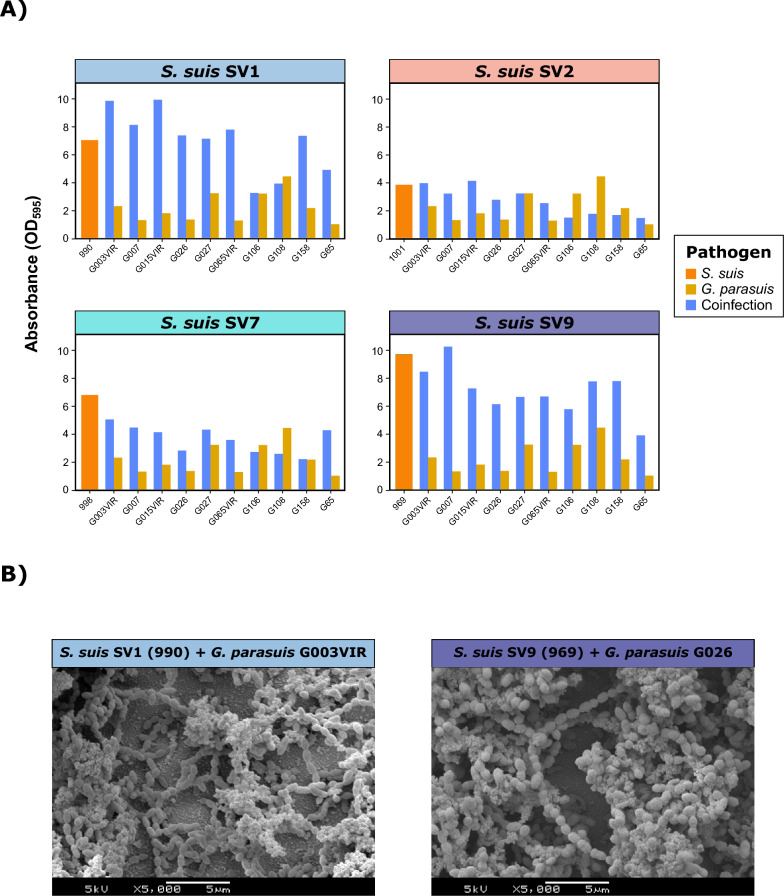
**Biofilm formation in coinfections of *Streptococcus suis* and *Glaesserella parasuis*.** **A)** Bar plots showing the quantitative biofilm formation (expressed as the absorbance OD_595_) of one representative *S. suis* isolate from each of the four main serovars (SVs) (orange), ten random *G. parasuis* isolates (green), and their coinfections (blue). **B)** Scanning electron microscopy (SEM) images of biofilm formation in coinfections between *S. suis* SV1 and *G. parasuis* G003 (left) and between *S. suis* SV9 (969) and *G. parasuis* G026 (right).

**Figure 7:**
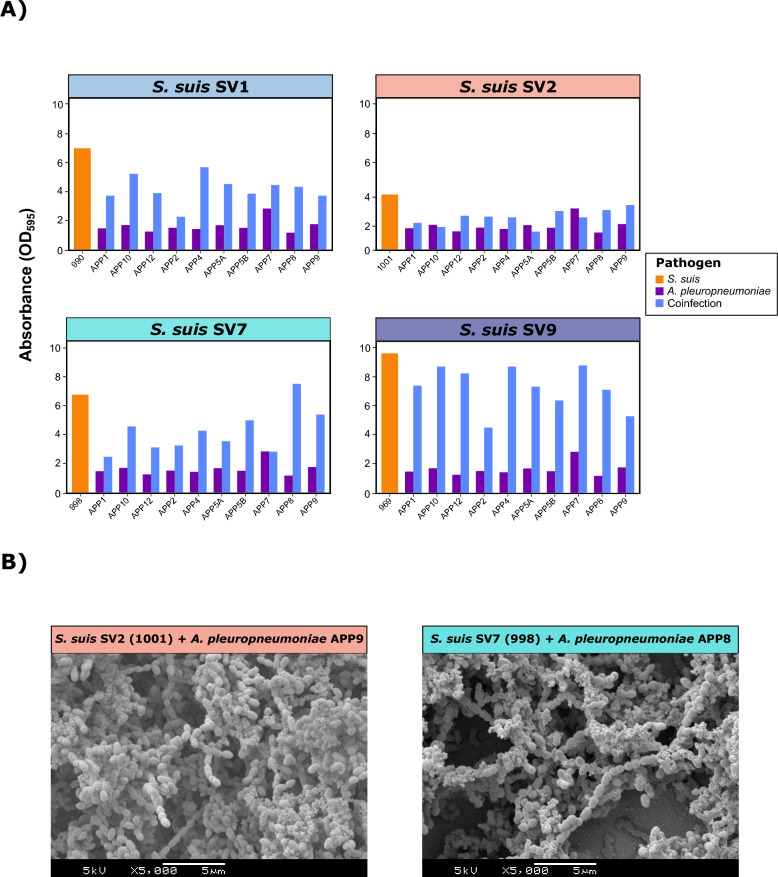
**Biofilm formation in coinfections of *Streptococcus suis* and *Actinobacillus pleuropneumoniae*.** **A)** Bar plots showing the quantitative biofilm formation (expressed as the absorbance OD_595_) of one representative *S. suis* isolate from each of the four main serovars (SVs) (orange), ten random *A. pleuropneumoniae* isolates (green), and their coinfections (blue). **B)** Scanning electron microscopy (SEM) images of biofilm formation in coinfections between *S. suis* SV2 (1001) and *A. pleuropneumoniae* APP9 (left) and between *S. suis* SV7 (998) and *A. pleuropneumoniae* APP8 (right).

Coinfections between *G. parasuis* and *S. suis* revealed an overall significant reduction in biofilm formation for *S. suis* for SV2 (*p*<0.05), SV7 (*p*<0.001), and SV9 (*p*<0.001) (Figure 6A). In contrast, *G. parasuis* increased its biofilm production in all SV coinfections, except for SV2, with notable increases in SV1 (*p*<0.001) and SV9 (*p*<0.001), nearly 3.5 times greater than the single *G. parasuis* DC average. Similar but more pronounced results were observed for *A. pleuropneumoniae* (Figure 7A). Coinfection increased biofilm formation in *A. pleuropneumoniae*, regardless of the *S. suis* SV (*p*<0.05), particularly for SV9 (DC increase of 4.5 times) and SV7 and SV1 (DC increase of 2.6 times). Conversely, all *S. suis* SVs presented a significant reduction in biofilm production (*p*<0.001). Notably, the only coinfection that increased biofilm formation was *S. suis* SV7 and *A. pleuropneumoniae* APP8. For both *G. parasuis* and *A. pleuropneumoniae* coinfections, SEM revealed that biofilm formation was determined mainly by *S. suis* (Figures 6B, 7B), with a reduced contribution of these pathogens to the biofilm matrix. These findings demonstrate that the associations between *S. suis* and other bacterial pathogens are not homogeneous and that substantial differences among SVs need to be considered.

A detailed description of the significant interactions between *S. suis* SVs and bacterial pathogens, including the effects on both *S. suis* and the bacterial pathogens, along with DC averages and potential increases, is available in Additional files 7 and 8.

## Discussion

*Streptococcus suis* is an opportunistic and zoonotic pathogen that naturally colonizes the respiratory tract in pigs [24]. Several factors, such as bacterial or viral coinfections and environmental stressors, can cause *S. suis* to transition from a commensal state to a pathogenic state [3]. Its pathogenesis involves several niche environments, with multiple virulence mechanisms, including VFs and biofilm formation [6]. Here, through extensive screening of virulence mechanisms in *S. suis* clinical isolates recovered from Spanish swine farms, we demonstrate that biofilm-forming ability is a significant pathogenic factor in certain *S. suis* SVs, particularly in those isolates harboring fewer virulence genes. Furthermore, *S. suis* interactions in biofilm formation with bacteria involved in the PRDC clearly vary among SVs and pathogens.

The multifactorial nature of *S. suis* pathogenicity was evident in our study, which revealed substantial diversity in the frequency of VFs among isolates, with 27 distinct gene patterns observed. Approximately one-third of the isolates (33.8%) carried all five evaluated VFs. Among these genes, the *mrp*, *epf*, and *sly* genes, which are frequently associated with virulence [25], were simultaneously present in 37.9% of the clinical *S. suis* isolates. Additionally, 86.7% and 66.7% of them harboured one or two of these genes, respectively. This finding aligns with previous studies suggesting that their absence is more commonly associated with *S. suis* isolates from healthy or carrier pigs in Europe and Asia [26,28]. Nonetheless, the absence of one or more of these VFs does not necessarily correlate with a lack of virulence [5, 29]. For example, *sly* is typically absent in isolates from North America, but these isolates do not exhibit reduced virulence compared with *sly* producing *S. suis* [30]. The complexity of VF associations is further highlighted by the relationship between VFs and *S. suis* SVs, as recently described [31]. This finding is particularly notable for SV7, in which a negative association with the presence of *epf* and *sly* was observed, which is consistent with a previous study on *S. suis* SV7 clinical isolates from pig farms in Germany [32]. These findings underscore the importance of considering other mechanisms, such as biofilm formation, when evaluating *S. suis* pathogenicity.

Biofilm formation is an essential pathogenic mechanism in *S. suis*, enabling its establishment in pig tissues, as most isolates can form biofilms [7]. Indeed, the development of bacterial meningitis is strongly associated with biofilm formation [33]. However, differences exist among strains. In this study, we demonstrated that in vitro biofilm formation is strongly influenced by *S. suis* SV. A previous investigation revealed differences in biofilm-forming ability between SV2 and SV9 isolates [23], and our wide-range assessment of 16 different Spanish *S. suis* SVs expanded on this finding. *S. suis* SV2 was the only SV categorized as having low biofilm-forming ability. Despite its prevalence in swine infections [34] and its role as a primary lineage in human infections worldwide [35], our study shows that, interestingly, biofilm formation is not an essential pathogenic factor for SV2, in contrast with other SVs. For example, *S. suis* SV9, an important and prevalent SV causing invasive disease in pigs in Europe [36], demonstrated strong biofilm formation regardless of its virulence gene arsenal.

Reduced virulence has been described as an important characteristic of biofilm infection in *S. suis* [37]. Our research revealed an overall significant reduction in biofilm formation among *S. suis* isolates harbouring the *epf*, *mrp*, and *sly* genes. Previous studies have shown differential expression of virulence genes under planktonic and biofilm conditions [37], which could explain the presence of virulent strains in the host respiratory tract as commensals. In addition, *S. suis* in a biofilm state is less likely to trigger the immune system [38]. Although *luxS* is involved in the LuxS/AI-2 quorum sensing (QS) system, a crucial regulatory network influencing biofilm formation [39], no significant differences in biofilm formation were detected when isolates harboring *luxS* were evaluated. This could be due to its high prevalence in clinical *S. suis*, underscoring its importance as a virulence determinant [40]. Interestingly, when we analysed the association between biofilm formation and VFs caused by SV, we found that the overall differences were reduced, likely due to the overrepresentation of *S. suis* SV2 isolates positive for most of the evaluated VFs. While this may represent a potential limitation, the analysis by serotype still revealed consistent patterns, such as significantly lower biofilm formation in the *epf*- and *sly*-positive SV2 and SV9 isolates, respectively. Notably, *S. suis* SV1 isolates carrying the *epf* gene exhibited significantly greater biofilm formation, which aligns with studies suggesting that highly pathogenic strains may exhibit strong biofilm formation [37]. These differences likely depend on specific gene expression patterns in the biofilm state rather than the mere presence of the genes. Therefore, further investigations are necessary to evaluate gene expression changes between planktonic and biofilm-forming cells among SVs with varying biofilm-forming abilities.

Other bacterial pathogens have also been shown to form biofilms within the PRDC [41], and their potential role in coinfections with *S. suis* should be considered. Here, we demonstrated the in vitro biofilm formation abilities of primary (*A. pleuropneumoniae*) and secondary (*G. parasuis* and *P. multocida*) PRDC pathogens recovered from Spanish swine farms. However, the biofilms formed by these bacterial pathogens were generally less robust than those formed by most *S. suis* SVs, except for *S. suis* SV2. The low to medium biofilm formation observed in *A. pleuropneumoniae* aligns with previous studies showing the biofilm-forming ability of most field isolates [42], since this pathogen is known to form biofilms in the lungs [43].

For *G. parasuis*, we observed a wide range of biofilm-forming abilities among our isolates, regardless of their virulence, with overall medium biofilm formation. Recently, 76 genes have been identified as potentially involved in *G. parasuis* biofilm formation. Nonetheless, differences were observed among isolates, even within the same SV, likely due to its open pangenome and variations in the accessory genome [44]. Compared with other PRDC pathogens, *P. multocida* was identified as an intermediate biofilm producer, with 65% of clinical isolates characterized as mid-level biofilm producers, which is consistent with recent findings [45]. An inverse association between capsular polysaccharide production and biofilm formation has been described, with encapsulated *P. multocida* isolates presumed to be more virulent and producing less biofilm than those with reduced capsular polysaccharide [46]. However, we could not corroborate this finding due to the limited *P. multocida* isolates used in the study and the fact that all of them were clinical and produced capsular polysaccharides. Since *P. multocida* was not the primary focus of this study, our results did not explore these differences in depth; hence, further investigations are needed.

Although *S. hyicus*, the causative agent of exudative epidermitis, is not regarded as a constituent of the PRDC, recent studies have described its potential role in swine respiratory disease cases [47, 48], leading us to consider the biofilm persistence of bacterial pathogens involved in the PRDC. In this study, the strong biofilm formation capacity of *S. hyicus* was confirmed, with more than 90% of the isolates showing robust biofilm formation, several of which were well above the threshold to be considered strong biofilm formers. To the best of our knowledge, this is the first report specifically addressing the biofilm formation of *S. hyicus*, although previous studies have evaluated its formation within sets of coagulase-negative staphylococci, yielding disparate results [49]. The strong biofilm formation observed in this study could serve as a starting point for future studies aimed at understanding the mechanisms of *S. hyicus* biofilm formation and its impact on the microbial environment in the respiratory tract.

Given the high prevalence of bacterial coinfections in the PRDC [50], mixed biofilms may be common and contribute to increased bacterial survival through interspecific competition, communication, and cooperation [8]. However, potential interactions involving *S. suis* have rarely been studied, with a primary focus on *S. suis* SV2, which was shown to be the lowest biofilm former among *S. suis* SVs. This research revealed that the contribution of each pathogen to in vitro biofilm formation differs depending on the bacterial pathogen and the *S. suis* SV involved. Notably, this is the first report to show that biofilm formation was stronger in mixed infections with *S. suis* and *P. multocida* than in single infections for both microorganisms, with a clear presence of both pathogens in the biofilm matrix. Additionally, we demonstrated an overall promotion of biofilm formation for *G. parasuis* and *A. pleuropneumoniae* when they were coinfected with *S. suis*, although it was determined mainly by the presence of *S. suis*. Thus, *S. suis* could contribute to the persistence of these pathogens integrated in the biofilm matrix. These findings are consistent with previous studies that analysed the role of *S. suis* in the persistence of both *G. parasuis* [51] and *A. pleuropneumoniae* [52] in mixed biofilms with *S. suis* SV2. In the case of *A. pneumoniae*, biofilm growth is promoted under hostile conditions, such as the absence of NAD, when coinfected with *S. suis* [53], revealing that mixed infections may be more difficult to eradicate. In contrast, *S. suis* coinfection with *S. hyicus* revealed that the latter was the main determinant in the biofilm matrix, highlighting its potential relevance in the persistence of *S. suis* in the respiratory tract, since both are frequently present in tonsils [54]. Understanding the role of biofilm formation in respiratory mixed infections will contribute to the establishment of optimal control measures for the PRDC, a syndrome that causes significant economic losses in pig production worldwide [55].

This study highlights the heterogeneity in virulence factors and in vitro biofilm formation among *S. suis* clinical isolates from Spanish swine farms, particularly those influenced by SV variations. Our findings indicate that while some *S. suis* SVs, such as SV2, have low biofilm-forming abilities, others, such as SV1, SV7 or SV9, exhibit robust biofilm formation, independent of their virulence gene arsenal. Additionally, the present study underscores the complexity of mixed biofilm formation in coinfections, revealing heterogeneous biofilm production in interactions between *S. suis* and other primary or secondary PRDC bacterial pathogens, such as *P. multocida*, *G. parasuis*, or *A. pleuropneumoniae*. Remarkably, *S. hyicus*, which is typically not associated with PRDC, displayed strong biofilm formation, suggesting its potential role in *S. suis* persistence in the upper respiratory tract. These insights pave the way for more detailed investigations into the mechanisms underlying biofilm formation and maintenance in PRDC-associated pathogens, ultimately contributing to the development of effective control measures to mitigate their economic impact in the swine industry.

## Supplementary Information

**Additional file 1:** Summary of bacterial isolates recovered from Spanish swine farms used in the study.

**Additional file 2: ** Combination of virulence factor (VF) genes in *Streptococcus suis* isolates recovered from Spanish swine farms.

**Additional file 3: ** Combination of virulence factor (VF) genes in *Streptococcus suis* isolates recovered from Spanish swine farms, categorized by serovar (SV).

**Additional file 4:** Significant associations (Fisher's exact test) between *Streptococcus suis* serovars (SVs) and the carriage of virulence factor (VF) genes (*p* < 0.05) were detected.

**Additional file 5:** Significant differences (Wilcoxon rank-sum test) in biofilm formation among *Streptococcus suis* serovars (SVs) (*p* < 0.05).

**Additional file 6: ** Significant differences (Wilcoxon rank-sum test) in biofilm formation among bacterial pathogens involved in the porcine respiratory disease complex (PRDC) and *Streptococcus suis* belonging to different serovars (SVs) (*p* < 0.05).

**Additional file 7: **Significant modification (Wilcoxon rank-sum test) in the biofilm formation of *Streptococcus suis* from different serovars (SVs) alone and in coinfection with bacterial pathogens involved in the porcine respiratory disease complex (PRDC) (*p* < 0.05).

**Additional file 8:** Significant modification (Wilcoxon rank-sum test) in the biofilm formation of bacterial pathogens involved in porcine respiratory disease complex (PRDC) alone and in coinfection with *Streptococcus suis* from different serovars (SVs) (*p* < 0.05).

## Data Availability

The datasets used and/or analysed during the current study are available from the corresponding author on reasonable request.

## Abbreviations

EF: extracellular factor
Exh: exfoliative toxin
FBS: fetal bovine serum
GAPDH: glyceraldehyde-3-phosphate dehydrogenase
LuxS: S-ribosylhomocysteinase
MRP: muramidase-released protein
NAD: nicotinamide adenine dinucleotide
OD: optical density
PBS: phosphate-buffered saline
PCA: principal component analysis
PCR: polymerase chain reaction
PRDC: porcine respiratory disease complex
SEM: scanning electron microscopy
SV: serovar
Tbp: transferring-binding protein
THB: Todd-Hewitt broth
VF: irulence factor
